# Pharmacological vs. non-pharmacological interventions for anxiety control in dental implant surgery: a systematic review

**DOI:** 10.3389/fdmed.2026.1891238

**Published:** 2026-07-09

**Authors:** Srilakshmi Mahadevan, Mamatha Shekar Shetty, Amitha Ramesh, Amanchi Vineela

**Affiliations:** Department of Periodontology, AB Shetty Memorial Institute of Dental Sciences (ABSMIDS), Nitte (Deemed to be University), Mangalore, India

**Keywords:** dental anxiety, dental implants, pharmacological management, non-pharmacological management, anxiolytics, sedation

## Abstract

**Background:**

Dental anxiety associated with dental implant surgery can adversely affect patient comfort, cooperation, and overall procedural outcomes. Both pharmacological and non-pharmacological interventions are commonly employed to manage preoperative anxiety; however, their comparative effectiveness remains unclear. A systematic evaluation of these approaches is important to determine their relative benefits in terms of anxiety reduction, onset of action, safety profile, and patient acceptability. Existing evidence suggests that pharmacological interventions may provide rapid anxiolytic effects, whereas non-pharmacological approaches, such as relaxation techniques, mindfulness-based interventions, and audio distraction, may reduce anxiety without the potential adverse effects associated with medication use. Therefore, this systematic review aims to compare the effectiveness of pharmacological and non-pharmacological anxiety control strategies in dental implant surgery and evaluate their clinical applicability in optimizing patient management.

**Methods:**

A comprehensive literature search was conducted across PubMed, Scopus, Web of Science, and the Cochrane Library to identify randomized controlled trials evaluating the effectiveness of pharmacological and non-pharmacological interventions for the management of dental anxiety in adult patients undergoing dental implant surgery, up to September 2025. The systematic review was conducted according to a pre-specified protocol in accordance with the Preferred Reporting Items for Systematic Reviews and Meta-Analyses (PRISMA) guidelines. The methodological quality and risk of bias of the included studies were assessed using the revised Cochrane Risk of Bias tool (RoB 2.0).

**Results:**

The electronic database search identified a total of 225 articles related to anxiety management during dental implant placement. By evaluating titles, abstracts, and full texts, 15 articles met the inclusion criteria; however, no relevant studies were found through hand searching. The evidence suggested that that multimodal anxiety-reduction strategies, particularly dexmedetomidine sedation and behavioural distraction techniques, are effective in reducing dental anxiety during implant procedures

**Conclusion:**

Both non-pharmacological and pharmacological anxiety management strategies demonstrate considerable potential in reducing dental anxiety during implant placement. Behavioural interventions, including effective communication, relaxation techniques, distraction methods, and complementary therapies, appear beneficial for patients with mild to moderate anxiety, whereas pharmacological sedation is particularly effective for managing moderate to severe dental fear, thereby enhancing patient cooperation, procedural comfort, and overall acceptance of implant therapy.

**Systematic Review Registration:**

https://www.crd.york.ac.uk/PROSPERO/view/CRD420261376502, identifier CRD420261376502.

## 1 Introduction

Corah (1969) introduced the definition of dental anxiety as patient stress manifested during dental visits (1). Dental trait anxiety includes both a chronic avoidance of dental visits and short-term emotional responses like fear and distress during procedures, often resulting from past negative experiences (2). Ranking as the fifth leading anxiety type, dental anxiety affects roughly 70% of implant patients with moderate-to-high preoperative levels, such anxiety may precipitate unfavourable physical and psychological responses throughout the treatment process (3). In anxiety disorders, persistent fear and anxiety emerge in response to situations ranging from vaguely outlined to sharply defined. Patients often display intense avoidance tactics or bear the stimuli with considerable fear and unease (4).

The diverse nature of anxiety disorders suggests that genetic, environmental, and other factors play differing roles in their development. Panic disorder, for example, has a more pronounced genetic component than many others (National Institute of Mental Health [NIMH], 1998), even if the exact genes are unknown. Others appear more closely tied to stressful life experiences (5). Inherited genetic predispositions in individuals with specific phobias like odontophobia may increase their overall risk for anxiety or targeted phobias. While the phobia of dentistry is not directly inherited, such genetic elements can interplay with diverse etiological contributors to foster its development (6).

Dental anxiety has a multifactorial etiology, arising from a complex interaction between external influences—such as traumatic past experiences, negative social conditioning, sensory stimuli within the dental environment, and ineffective communication—and intrinsic patient-related factors, including personality traits, temperament, cognitive characteristics, sociodemographic background, oral health status, and medical comorbidities. A clear understanding of these contributing factors enables clinicians to identify anxious patients and implement appropriate management strategies effectively (7).

On a global level, dental anxiety remains a widespread public health concern, with studies reporting rates of about 4% to 23% in Europe, 20% to 30% in Indonesia, 10 to 12 million affected individuals in the United States, and 35 million people experiencing dental anxiety overall; higher rates have also been reported in South America and Ecuador, where severe anxiety has reached 39% in some studies. Evidence also shows that many anxious patients delay dental care, with one report finding that individuals with dental phobia waited an average of 17.3 days in pain before seeking treatment, highlighting the need for early recognition and management (8).

**Figure 1:**
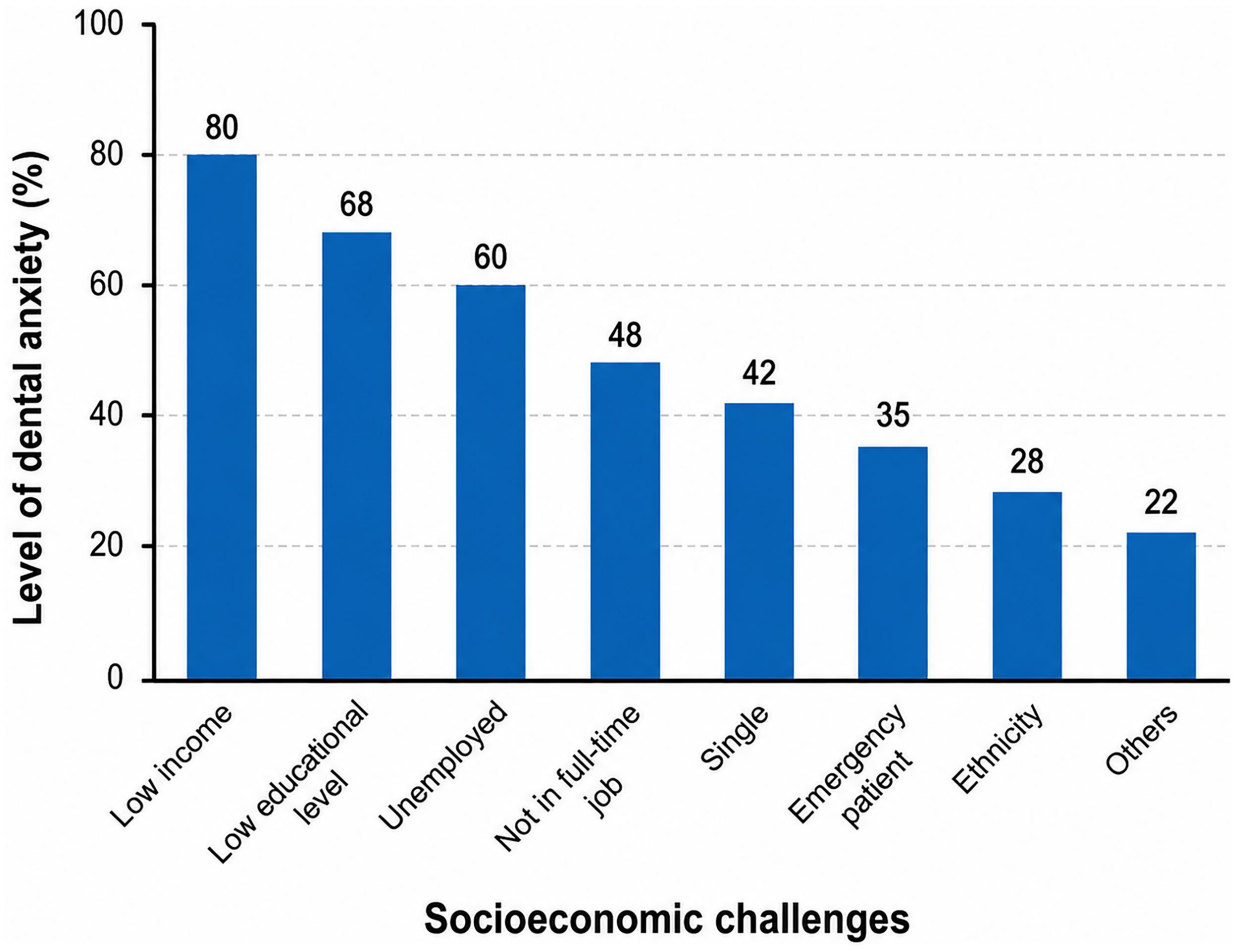
Common sources of dental anxiety among patients undergoing dental procedures.

Dental anxiety is closely linked to poorer oral health outcomes. Research has shown that avoiding dental treatment is strongly associated with higher anxiety levels as well as increased caries experience and DMFS scores. Highly anxious individuals are also more likely to miss dental appointments, attend irregularly, or completely avoid dental care. Those with significant anxiety may also be more likely to cancel, postpone, or fail to schedule appointments in the first place. This pattern of avoidance contributes to a greater burden of dental decay and a higher need for extensive oral rehabilitation (9). Figure 1. Illustrates levels of anxiety associated with different socioeconomic challenges (10).

**Figure 2:**
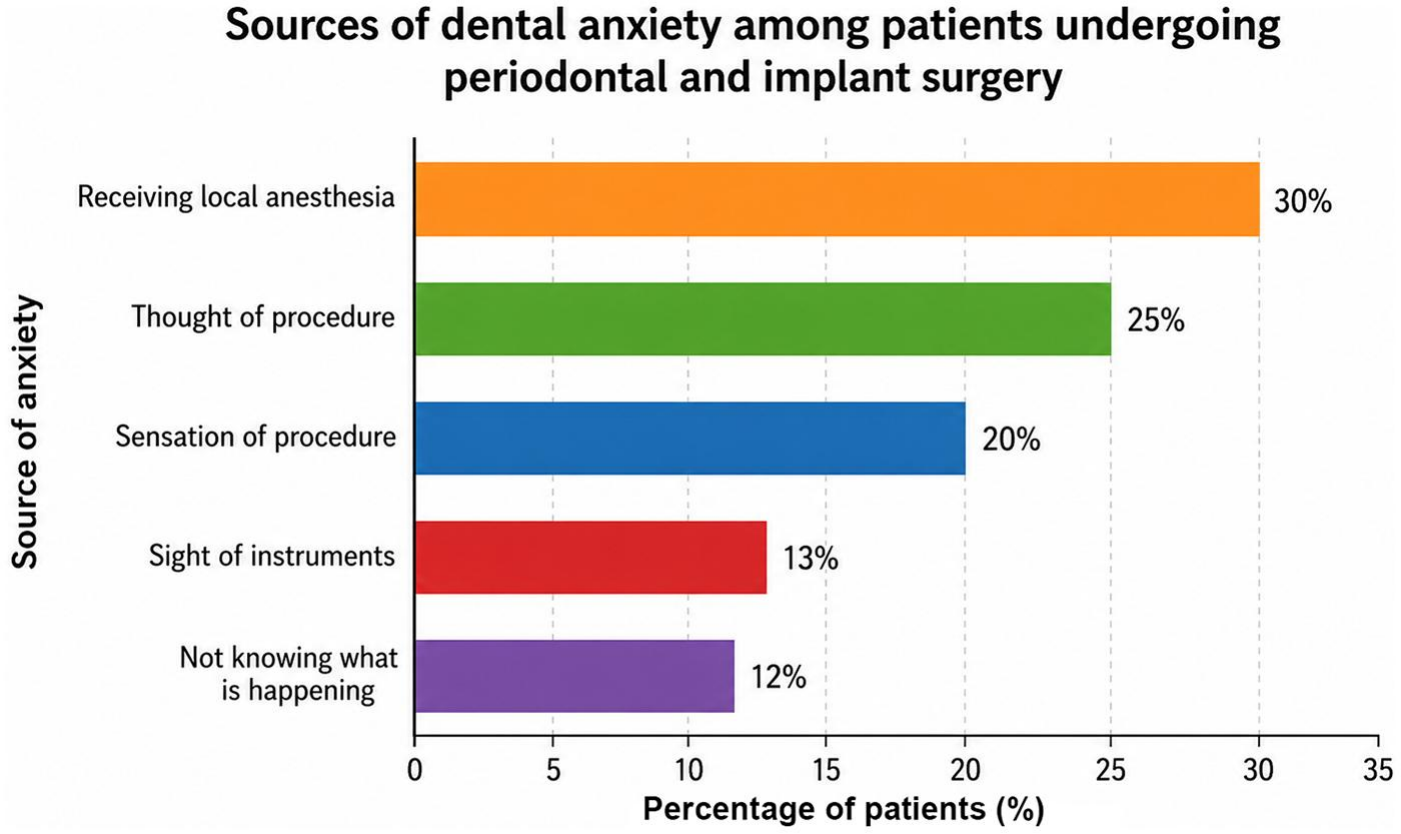
Illustrates patient-reported sources of anxiety associated with periodontal and implant surgical procedures.

Dental implants represent a breakthrough in oral rehabilitation (11). In dentistry, dental implants provide a robust and lasting solution to address edentulism effectively with marked success rates and improvements in oral functionality, aesthetics and oral health quality of life (12). In developing countries, despite of their benefits dental implants are seldom selected and influenced by several factors that affects the choice (13). Technological progress has eased some anxieties around dental implant treatments dental anxiety lingers in segments of society and is still considered as an underexplored area (14). Over almost five decades, developments in biomaterials, biologic understanding, and implant dentistry research have established implant-supported prostheses as the preferred treatment for most edentulous conditions, which yield 97%–99% success rates and remarkable durability when implants are ideally positioned, prosthetically optimized, and routinely maintained (15). A patient who visits a clinic for dental implant surgery has numerous expectations about the treatment, including dread of discomfort during the process. Implant implantation may cause mild to severe stress, and effective anxiety management may be linked to pain relief (16). Figure 2. Patient-reported sources of anxiety associated with periodontal and implant surgical procedures (17).

During implant therapy, anxiety is shown to have a major impact on both physiological and psychological outcomes. Stress-induced alterations in blood parameters and altered hemodynamic can have an indirect impact on haemoglobin levels. When paired with increased anxiety, patients with decreased haemoglobin levels may already have impaired oxygen-carrying ability, which can worsen fatigue, delayed healing, and general discomfort throughout the perioperative period. As a result, preoperative anxiety, haemoglobin levels, and the surgical experience are all interrelated and have a significant impact on patient satisfaction. In addition to making pain and unpleasant feelings during implant insertion more apparent, more anxiety may also have a detrimental effect on healing results, which would lower overall satisfaction (17). Anxiety can cause patients to be uncooperative during implant surgery, which prolongs the procedure and ultimately contributes to patient dissatisfaction (18).

Dental anxiety may be addressed with either pharmacological or nonpharmacological or combination of these (19). In general, dental anxiety can be treated with either of these techniques and it depends on the patient features, clinical circumstances, the dentist's training and experience, and the level of dental anxiety (20). To lessen anxiety and increase compliance during dental treatment, a few non-pharmacological behaviour management strategies have been suggested. Voice control, tell-show-do, positive reinforcement, distraction, nonverbal communication, hand-over-mouth technique (HOM), hand-over-mouth with airway restriction (HOMAR), physical restraint, contingent escape, contingent distraction, relaxation training, hypnosis, are some of these strategies. To make the dentist visit more comfortable and safer for children, additional supportive techniques like magic tricks, positive imagery, and environmental changes have also been used (21).

The volume of research assessing pharmacological and non-pharmacological interventions in managing anxiety associated with dental implant therapies is expanding, although the evidence is still inconsistent and conflicting. To further improve patient comfort and surgical outcomes, a number of studies have investigated on non-pharmacological as well. However, these studies varied significantly in terms of research design, intervention regimens, outcome measures, and evaluation techniques, making direct comparison challenging. Furthermore, rather than offering a comparative assessment between pharmacological and non-pharmacological treatments, most previous systematic reviews have concentrated on individual modalities or certain medication classes.

In the context of dental implant surgery, there is currently no thorough systematic review that critically synthesizes evidence to ascertain the relative efficacy of these two broad techniques. Thus, the purpose of this systematic review is to assess and contrast the effectiveness of pharmacological and non-pharmacological dental implant placement therapies, with a focus on patient-centred outcomes including overall satisfaction, anxiety reduction, and pain perception. This study aims to elucidate their relative advantages and support evidence-based clinical decision-making by including current research.

## 2 Materials and methodology

### 2.1 Protocol and registration

This systematic review was reported in accordance with the Preferred Reporting Items for Systematic Reviews and Meta-Analyses (PRISMA) statement. The review protocol was registered in the PROSPERO database with the registration number CRD420261376502.

### 2.2 Research question

In adult patients with dental anxiety undergoing dental implant therapy, what is the comparative effectiveness of pharmacological and non-pharmacological anxiety management interventions in reducing associated dental anxiety and how do these interventions influence the secondary parameters such as pain perception, patient comfort, physiological parameters, sedation level and satisfaction?

### 2.3 PICO framework

Population (P): Adult patients (≥18 years) with dental anxiety undergoing dental implant therapy or implant placement procedures.

Intervention (I): Pharmacological anxiety control strategies employed during dental implant therapy.

Comparator (C): Non-pharmacological anxiety control strategies employed during dental implant therapy.

Outcomes (O): Primary outcome: Reduction in dental anxiety and stress levels among patients undergoing dental implant therapy assessed using validated anxiety assessment tools.

Secondary outcomes: Conscious sedation depth and effectiveness, postoperative analgesia and pain perception, patient satisfaction, surgeon satisfaction, and changes in physiological parameters including vital signs and hemodynamic responses.

### 2.4 Eligibility criteria

Studies were considered eligible for inclusion if they met the following criteria: (1) randomized controlled trials (RCTs); (2) involved adult patients (≥18 years) undergoing dental implant placement for the rehabilitation of edentulous spaces; (3) evaluated pharmacological and/or non-pharmacological interventions specifically intended to reduce dental anxiety before, during, or immediately after implant surgery; (4) reported at least one relevant outcome related to anxiety reduction, including validated anxiety scales (State-Trait Anxiety Inventory (STAI), Modified Dental Anxiety Scale (MDAS), Visual Analogue Scale (VAS), physiological parameters (heart rate, blood pressure, oxygen saturation, salivary cortisol), pain perception, patient satisfaction, surgeon satisfaction, or sedation outcomes; and (5) were published as full-text articles in peer-reviewed journals in the English language.

Studies were excluded if they were non-randomized studies, observational studies, cohort studies, case-control studies, case series, case reports, review articles, systematic reviews, meta-analyses, conference abstracts, editorials, letters to the editor, expert opinions, animal studies, *in vitro* investigations, or other non-original research publications. Studies that did not involve dental implant surgery, did not evaluate anxiety-management interventions, lacked relevant outcome data, included participants younger than 18 years, were not available as full-text articles, or were published in languages other than English were also excluded. The reasons for exclusion of studies at the full-text screening stage are presented in Table 1.

**Table 1:** Summary of few excluded studies and reasons for exclusion, after accessing full text.

Study (Author & Year)	Country	Objective of study	Reason for exclusion
Ganga Mohan et al. (2023) (45)	India	To evaluate psychological and physiological relaxation using yoga during dental implant surgery	Full text unavailable
Stephan Eitner et al. (2014) (44)	Germany	To assess effectiveness of audio pillow with hypnotherapy/music for anxiolysis during implant surgery	Non-English language
Hiroyoshi Kawaai et al. (2014) (46)	Japan	To compare IV sedation protocols using dexmedetomidine or propofol during implant surgery	Full text unavailable
Peng Li et al. (2020) (47)	China	To evaluate different doses of dexmedetomidine and midazolam in implant surgery	Chinese language article
Eleftherios Martinis (2013) (48)	Greece	To review management strategies for dental anxiety in implant dentistry	Non-clinical study
Pourabbas et al. (2022) (39)	Iran	To evaluate effect of conscious sedation on anxiety reduction in implant surgeries	Non-clinical study

### 2.5 Information sources and search strategy

**Figure 3:**
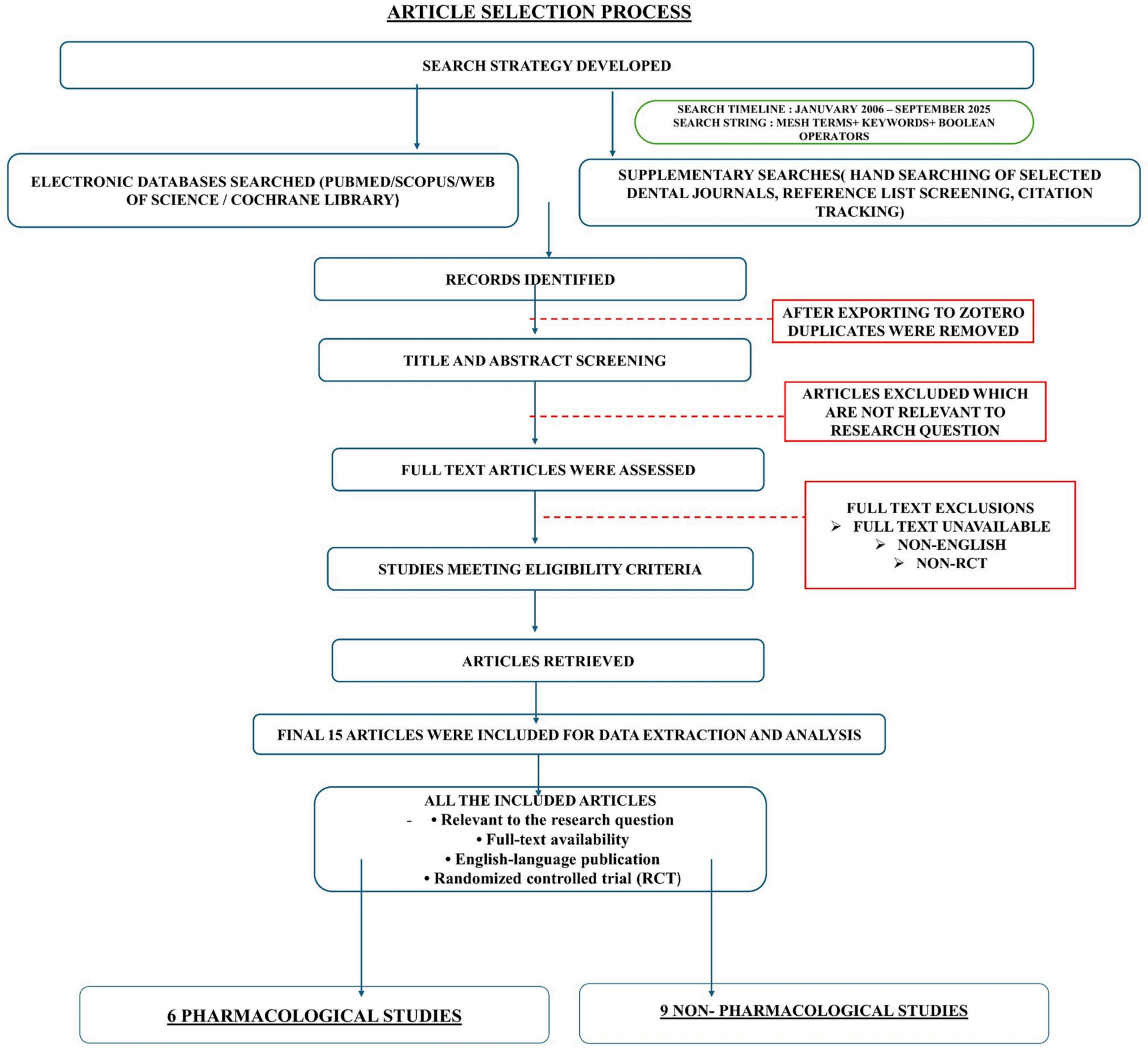
Article selection process and categorization of included studies.

**Figure 4:**
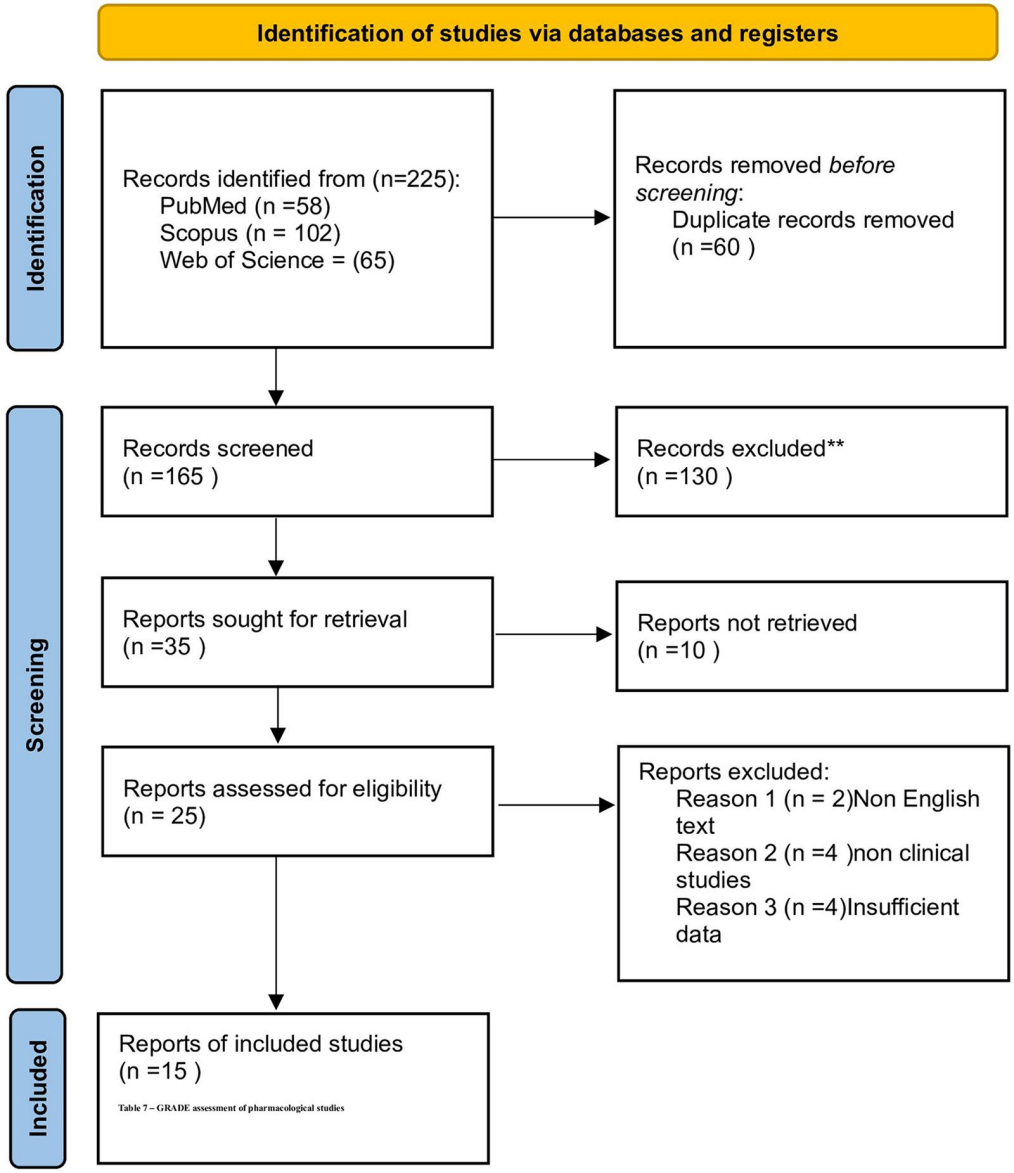
PRISMA 2020 flow diagram of study selection.

A systematic literature search was conducted in PubMed, Scopus, Web of Science, and the Cochrane Library to identify relevant studies published between January 2006 and September 2025. The search strategy was developed using a combination of Medical Subject Headings (MeSH) terms, free-text keywords, and Boolean operators (AND, OR), and is summarized in Table 2. The initial search strategy was formulated for PubMed and subsequently adapted for Scopus, Web of Science, and the Cochrane Library according to the indexing systems and search functionalities of each database. The article selection process, including study identification, screening, eligibility assessment, and final inclusion, is presented in Figure 3. The final electronic search was performed on 30 September 2025. All retrieved records were exported to Zotero reference management software, where duplicate citations were identified and removed. Following deduplication, titles and abstracts were screened independently by two reviewers according to predefined inclusion and exclusion criteria. Studies considered potentially eligible underwent full-text assessment, and any disagreements regarding study selection were resolved through discussion and consensus. In addition to electronic database searching, supplementary searches were performed through manual screening of the reference lists of all included studies and relevant review articles to identify additional eligible studies. Furthermore, hand-searching of selected dental journals was undertaken to ensure literature saturation and to retrieve studies that may not have been indexed in the electronic databases. The study selection process is presented in the PRISMA 2020 flow diagram Figure 4.

**Table 2:** Search strategy.

Databases	Search terms
PubMed	[“Dental Anxiety”(Mesh) OR dental anxiety* OR dental fear* OR odontophobia* OR preoperative anxiety*] AND [“Dental Implants”(Mesh) OR dental implant surgery* OR implant placement* OR implant dentistry*] AND (pharmacological intervention* OR sedation OR conscious sedation OR anxiolytic agents OR dexmedetomidine OR midazolam OR remimazolam OR analgesics OR non-pharmacological intervention* OR music therapy OR mindfulness OR virtual reality OR distraction therapy OR behavioral intervention* OR patient education*)
Scopus	(“dental anxiety” OR “dental fear” OR odontophobia OR “preoperative anxiety”) AND (“dental implants” OR “implant surgery” OR “implant placement”) AND (“pharmacological intervention” OR sedation OR anxiolytics OR dexmedetomidine OR midazolam OR remimazolam OR “non-pharmacological intervention” OR “music therapy” OR mindfulness OR “virtual reality” OR distraction OR “behavioral intervention” OR “patient education”)
Cochrane Central	(“dental anxiety” OR “dental fear”) AND (“dental implants” OR “implant surgery”) AND (sedation OR anxiolytics OR “music therapy” OR mindfulness OR “virtual reality” OR distraction OR “behavioral intervention”)
Web of Science	TS = (“dental anxiety” OR “dental fear” OR odontophobia OR “preoperative anxiety”) AND TS = (“dental implants” OR “implant surgery” OR “implant placement”) AND TS = (sedation OR anxiolytics OR dexmedetomidine OR midazolam OR remimazolam OR “music therapy” OR mindfulness OR “virtual reality” OR distraction OR “behavioral intervention” OR “patient education”) AND Humans
Boolean operators	AND/OR

### 2.6 Data collection

Three authors independently extracted data from each included study using the Rayyan® platform. The extracted variables included study characteristics (first author, publication year, study design, sample size, and follow-up duration), details of the interventions and comparators, outcome measures, and principal findings. Discrepancies in data extraction were resolved through discussion until consensus was achieved. A fourth author independently cross-checked the extracted data for accuracy and completeness. The final dataset was reviewed and approved by all authors prior to analysis.

## 3 Results

### 3.1 Selection of eligible studies included

Electronic searches conducted across PubMed, Scopus, Web of Science and Cochrane identified a total of 225 records. After removal of 60 duplicate records, 165 records remained for title and abstract screening. Of these, 130 records were excluded due to irrelevance to the research objective, non-clinical study design. Subsequently, 35 full-text reports were sought for retrieval, of which 10 reports could not be retrieved because of access limitations. A total of 25 full-text articles were assessed for eligibility. Among these, 10 studies were excluded for the following reasons: non-English language articles (*n* = 2), non-clinical studies (*n* = 4), and insufficient clinical data (*n* = 4). Finally, 15 studies met the predefined inclusion criteria and were included in the qualitative analysis.

### 3.2 Intervention characteristics of eligible studies

A total of 15 randomized controlled trials (RCTs) published between 2012 and 2025 met the eligibility criteria and were included in this systematic review. Of these, six studies evaluated pharmacological interventions for anxiety management during dental implant surgery, whereas nine studies investigated non-pharmacological approaches. The included studies comprised a total of 1,238 participants undergoing dental implant therapy, with 307 participants enrolled in pharmacological studies and 931 participants enrolled in non-pharmacological studies.

**Figure 5:**
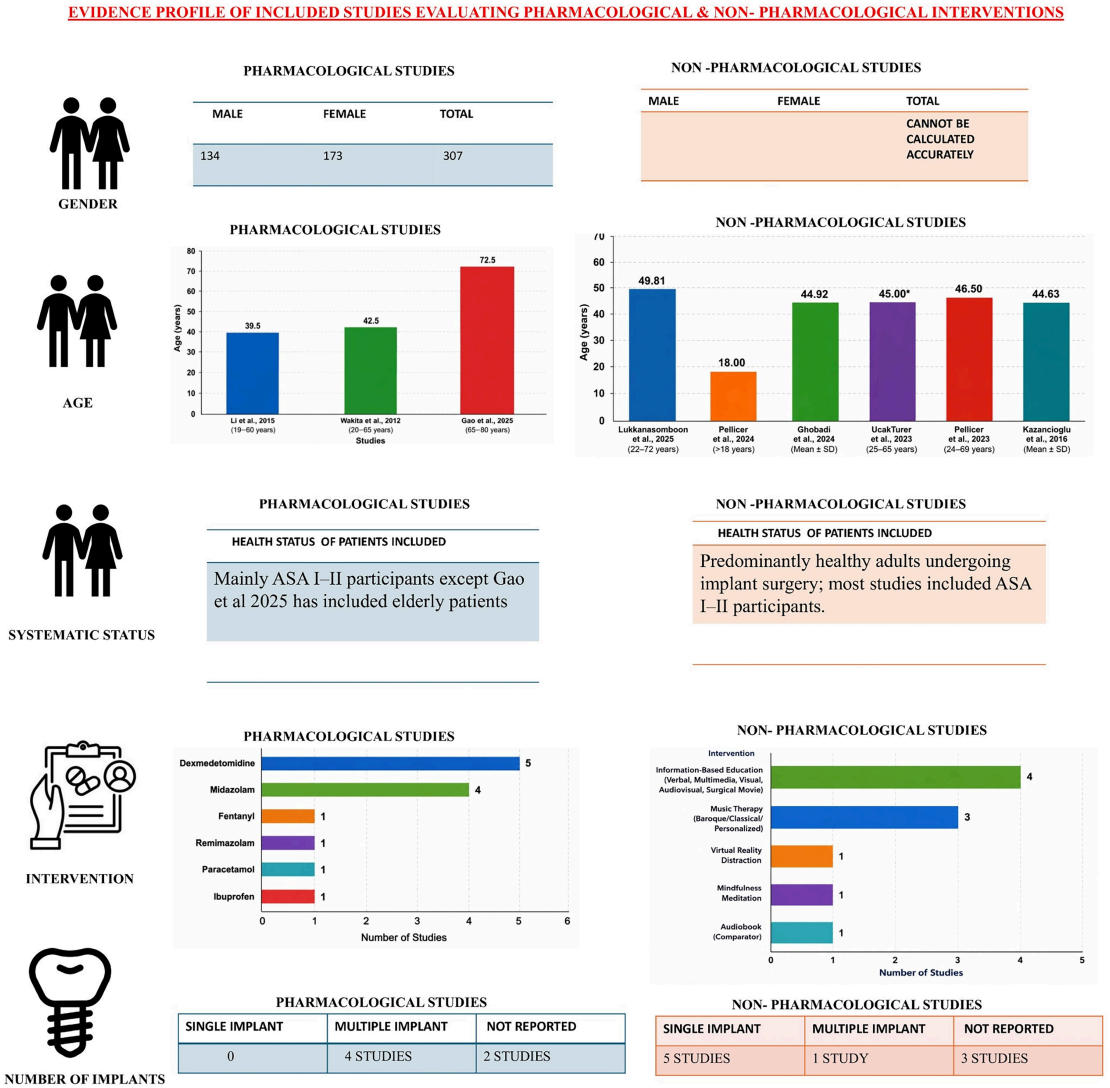
Evidence profile of included studies evaluating pharmacological and non-pharmacological interventions for dental anxiety during implant surgery.

The studies were conducted across a wide geographical distribution, predominantly in Asia and Europe. China and Spain contributed the highest number of studies, while additional investigations were conducted in Japan, Turkey, Iran, Thailand, Saudi Arabia, Egypt, and Canada. Publication years ranged from 2012 to 2025, reflecting growing research interest in anxiety management during implant therapy. The included studies consisted of one study published in 2012, one in 2015, one in 2016, one in 2017, one in 2019, two in 2020, one in 2021, one in 2022, one in 2023, two in 2024, and four in 2025. Figure 5 provides an overview of the characteristics of the included studies, highlighting participant demographics, health status, intervention modalities, and implant-related variables across both pharmacological and non-pharmacological anxiety management approaches.

Sample sizes varied considerably across studies, ranging from 24 to 300 participants, indicating substantial clinical heterogeneity. Most studies recruited systemically healthy adult patients (ASA I–II) undergoing either single-implant or multiple-implant rehabilitation procedures. A limited number of studies specifically focused on elderly patients receiving implant therapy. Both conventional delayed implant placement and immediate implant placement protocols were represented among the included studies.

Considerable heterogeneity was observed in the intervention protocols. Pharmacological studies primarily evaluated conscious sedation and anxiolytic strategies, including dexmedetomidine, midazolam, remimazolam, fentanyl-based regimens, paracetamol, and ibuprofen administered at varying dosages and routes of administration. The pharmacological interventions included remimazolam (0.05 mg/kg loading dose followed by 0.1–0.3 mg/kg/h infusion), dexmedetomidine (0.5–1 μg/kg loading/bolus dose followed by 0.2–1.0 μg/kg/h infusion), midazolam (0.05 mg/kg loading dose followed by 0.04–0.2 mg/kg/h infusion), paracetamol (1,000 mg), ibuprofen (600 mg), and dexmedetomidine–midazolam combination regimens. The pharmacological interventions were administered primarily via the intravenous route, including dexmedetomidine, midazolam, remimazolam, and fentanyl-based conscious sedation protocols. Oral administration was employed in one study evaluating paracetamol (1,000 mg) and ibuprofen (600 mg) for anxiety and stress reduction during implant surgery.

In contrast, non-pharmacological studies investigated a broad range of behavioral and distraction-based interventions, including music therapy, personalized music interventions, virtual reality distraction, mindfulness meditation, verbal counselling, written and multimedia educational materials, audiovisual information, and audiobook-based distraction. Outcome assessment methods also varied among studies and included validated anxiety scales, physiological parameters, pain scores, patient satisfaction measures, and sedation-related outcomes. The non-pharmacological interventions were administered at different stages of the implant treatment process. Educational interventions, including multimedia information, audiovisual information, verbal counselling, and surgical movie presentations, were delivered preoperatively before implant surgery. Mindfulness meditation was provided as a brief preoperative relaxation session. In contrast, distraction-based interventions such as virtual reality and music therapy, including personalized music programs, were administered during the implant surgical procedure.

The diversity of patient populations, implant protocols, anxiety-management interventions, outcome measures, and follow-up periods precluded direct comparison across studies and highlights the clinical heterogeneity of the available evidence.

In 43 implant patients, Wakita et al. (2012, Japan) assessed dexmedetomidine with and without midazolam. They found that the inclusion of midazolam improved amnesia and decreased intraoperative awareness, reducing procedural dread and anxiety during implant surgery (22).

In contrast Li et al. (2015, China) found that the dexmedetomidine group had significantly reduced pain scores, sedation scores, inflammatory markers (IL-6 and TNF-α), and oxidative stress markers when compared to midazolam + fentanyl in 60 patients. The study found that dexmedetomidine decreased the physiological stress reactions linked to dental anxiety in addition to improving sedation (23).

In 60 implant patients, Wang et al. (2020, China) found that dexmedetomidine produced better sedative quality, longer postoperative analgesia, and higher patient and surgeon satisfaction when compared to midazolam. The authors stressed that by encouraging deeper sedation and improved patient participation, dexmedetomidine alleviated anxiety (24).

Remimazolam was found to provide faster sedation onset, better hemodynamic stability, lower hypotension rates, and quicker recovery when compared to dexmedetomidine in 74 elderly implant patients by Gao et al. (2025, China). This suggests that remimazolam offers superior anxiety control in elderly patients undergoing implant rehabilitation (25).

When comparing paracetamol and ibuprofen during computer-guided flapless implant surgery, Adly et al. (2021, Egypt/Saudi Arabia) found that the paracetamol group had significantly lower salivary cortisol levels, suggesting better anxiety and stress reduction despite similar pain scores between the groups. Because dexmedetomidine-based sedation procedures have better anxiolysis, analgesia, and physiologic stability than traditional sedatives like midazolam, pharmacological studies have generally favoured them (26).

A prospective double-blind randomized controlled experiment was carried out in Turkey by Güldiken et al. (2021) to compare midazolam and dexmedetomidine for conscious sedation during dental implant surgery in 43 patients. Although midazolam showed a quicker onset of sedation, the study indicated that dexmedetomidine considerably reduced postoperative pain, increased patient satisfaction, decreased oxygen desaturation, and shortened the length of the procedure. Retrograde amnesia and patient preference for the sedative did not differ significantly. Overall, the authors concluded that for conscious sedation and anxiety control during dental implant procedures, dexmedetomidine is a safer and more effective substitute for midazolam (27).

When compared to pharmacological intervention, the results of the non-pharmacological investigations were more inconsistent.

A randomized clinical trial was carried out by Lukkanasomboon et al. (2025) to assess the effects of multimedia information on patient understanding, anxiety, and decision-making about computer-assisted and freehand dental implant surgery. According to the study, patients comprehension of implant operations was greatly enhanced by multimedia educational interventions, which also helped to lower preoperative anxiety (28).

Classical and baroque music dramatically lowered blood pressure, physiologic stress, and Modified Dental Anxiety Scale (MDAS) scores during implant insertion, according to Pellicer et al. (2023, Spain) and 2024, establishing music therapy as an easy and successful anxiety reduction technique (29, 30).

While UcakTurer et al. (2023, Turkey) observed that mindfulness meditation reduced cortisol levels, heart rate, blood pressure, and STAI-S anxiety scores, Ghobadi et al. (2024, Iran) reported that virtual reality distraction significantly reduced anxiety and pain scores by shifted patient attention away from surgery (31, 32). Personalized music therapies also decreased emotional tension, anxiety, and postoperative pain memory, according to Bertacco et al. (2022, Canada) (33). On the other hand, research assessing surgical video or audiovisual material yielded contradictory findings. While Camacho-Alonso et al. (2019, Spain) and Kazancioglu et al. (2016, Turkey) reported elevated anxiety levels in patients exposed to surgical videos or audiovisual presentations prior to implant surgery, Sghaireen et al. (2020, Saudi Arabia) discovered that direct verbal explanation reduced anxiety more effectively than visual information (34–36).

The findings presented here demonstrate that extensive procedural imagery may exacerbate dread and anticipatory stress in implant patients, even though soothing distraction strategies and encouraging verbal therapy effectively alleviate dental anxiety. Tables 3–6 summarize the characteristics and outcomes of the included pharmacological and non-pharmacological studies.

**Table 3:** Characteristics of included pharmacological studies.

Author & Year	Country	Study design	Sample size	Test intervention	Comparator	Outcomes evaluated
Gao et al., 2025 (25)	China	Prospective Parallel-Group Randomized Controlled Trial	74 elderly patients undergoing dental implant surgery	Remimazolam conscious sedation (0.05 mg/kg loading dose followed by infusion 0.1–0.3 mg/kg/h)	Dexmedetomidine conscious sedation (0.5 μg/kg bolus followed by infusion 0.2–1.0 μg/kg/h)	Incidence of hypotension, bradycardia, respiratory depression, MOAA/S score, sedation onset time, delayed recovery/discharge, hemodynamic and respiratory variables
Adly et al., 2021 (26)	Egypt/Saudi Arabia	Double-Blinded Randomized Crossover Clinical Trial	30 patients undergoing bilateral flapless dental implant surgeries	Paracetamol 1,000 mg before and after implant surgery	Ibuprofen 600 mg before and after implant surgery	Stress and anxiety (salivary cortisol), VAS pain score, postoperative swelling assessed using computer vision system
Güldiken et al., 2021 (27)	Turkey	Prospective Double-Blind Parallel Randomized Clinical Trial	40 patients undergoing dental implant surgery	Dexmedetomidine conscious sedation administered during implant surgery	Midazolam intravenous conscious sedation administered during implant surgery	Sedation level, pain score, patient satisfaction, surgeon satisfaction, oxygen saturation, respiratory depression, hemodynamic parameters, recovery profile
Wang et al., 2020 (24)	China	Parallel-Group Randomized Clinical Trial	60 patients undergoing dental implantation surgery	Dexmedetomidine sedation (1 μg/kg loading + infusion 0.2–0.7 μg/kg/h)	Midazolam sedation (0.05 mg/kg loading + infusion 0.04–0.2 mg/kg/h)	OAAS sedation score, VAS pain score, duration of postoperative analgesia, surgeon satisfaction, patient satisfaction, HR, BP, SpO_2_
Li et al., 2015 (23)	China	Randomized Parallel-Group Clinical Study	60 patients undergoing dental implant surgery	Dexmedetomidine + fentanyl intravenous sedation	Midazolam + fentanyl intravenous sedation	VAS pain score, OAAS sedation score, IL-6, TNF-α, SOD, MDA, SBP, HR, RR, RPP, SpO_2_
Wakita et al., 2012 (22)	Japan	Randomized Parallel-Group Clinical Trial	43 subjects undergoing dental implant surgery	Dexmedetomidine combined with midazolam using different dosing protocols	Dexmedetomidine alone and alternative dose regimens	Ramsay Sedation Score (RSS), amnesia, patient satisfaction, systolic/diastolic BP, HR, SpO_2_, intraoperative arousal response

**Table 4:** Clinical outcomes of included pharmacological studies.

Study	Intervention	Primary & secondary outcomes	Inference related to dental anxiety reduction
Gao et al., 2025 (China) (25)	**Remimazolam vs. Dexmedetomidine** for conscious sedation in elderly implant patients	**Primary outcome:** Incidence of hypotension. **Secondary outcomes:** Bradycardia, respiratory depression, sedation onset time, delayed awakening, delayed discharge, MOAA/S score, PADSS score, hemodynamic variables. **Results:** Hypotension incidence was lower in remimazolam group (5.4%) vs. DEX group (24.3%). Sedation onset was faster with remimazolam (105 ± 21 s vs. 599 ± 172 s). Delayed discharge was lower with remimazolam (2.7% vs. 16.2%). Both groups achieved satisfactory sedation with minimal respiratory depression.	Faster sedation onset and better hemodynamic stability with remimazolam reduced procedural anxiety in elderly patients. Patients became calm more rapidly and recovered quicker, improving overall comfort during implant surgery.
Adly et al., 2021 (Egypt/Saudi Arabia) (26)	**Paracetamol vs. Ibuprofen** during computer-guided flapless implant surgery	**Primary outcomes:** Salivary cortisol levels (stress/anxiety marker), VAS pain score. **Secondary outcomes:** Facial swelling measured using computer vision system. **Results:** Salivary cortisol was significantly lower in the paracetamol group (4.1 ± 1.08 ng/mL) compared with ibuprofen (6.2 ± 0.94 ng/mL). Pain scores were similar between groups (13.1 ± 1.1 vs. 12.9 ± 2.3). Swelling control was better with ibuprofen.	Paracetamol reduced dental anxiety more effectively by blunting emotional fear response and reducing stress hormone levels. Patients perceived less stress and fear associated with implant surgery even though pain scores remained similar.
Güldiken et al., 2021 (27)	**Dexmedetomidine conscious sedation vs. Midazolam conscious sedation** during dental implant surgery	**Primary outcomes:** Faster sedation onset with midazolam (10 ± 3.16 min) vs. dexmedetomidine (17.5 ± 2.99 min); additional sedative requirement lower with dexmedetomidine (9.1% vs. 42.9%). **Secondary outcomes:** Lower desaturation (4.5% vs. 52.4%), lower rescue analgesic use (22.7% vs. 85.7%), shorter surgery duration, lower pain scores, and higher patient satisfaction with dexmedetomidine.	Dexmedetomidine provided better anxiety control with improved analgesia, minimal respiratory depression, and greater patient comfort during implant surgery.
Wang et al., 2020 (China) (24)	**Dexmedetomidine vs. Midazolam** in dental implantation sedation	**Primary outcomes:** OAAS sedation score, VAS pain score, postoperative analgesia duration. **Secondary outcomes:** Surgeon satisfaction, patient satisfaction, HR, BP, SpO_2_, recovery profile. **Results:** Dexmedetomidine produced significantly lower OAAS scores and longer postoperative analgesia than midazolam. Surgeons reported significantly higher satisfaction with dexmedetomidine sedation. Better sedation quality and analgesia were observed with DEX.	Dexmedetomidine reduced dental anxiety more effectively by producing deeper sedation, better analgesia, and improved patient cooperation. Reduced awareness and prolonged analgesia decreased fear associated with implant procedures.
Li et al., 2015 (China) (23)	**Dexmedetomidine + fentanyl vs. Midazolam + fentanyl** for implant surgery sedation	**Primary outcomes:** VAS pain score, Observer's Assessment of Alertness/Sedation Scale (OAAS). **Secondary outcomes:** Plasma IL-6, TNF-α, SOD, MDA levels; SBP, HR, RR, RPP, SpO_2_. **Results:** VAS and OAAS scores were significantly lower in the DEX group than in the midazolam group. TNF-α, IL-6, and MDA levels were significantly lower at 2 h and 4 h in the DEX group. SOD levels were significantly higher in DEX patients. DEX group demonstrated better postoperative analgesia and less oxidative stress.	Dexmedetomidine reduced dental anxiety by producing deeper sedation and improved analgesia while suppressing inflammatory stress responses. Patients experienced less pain perception and greater calmness during implant surgery.
Wakita et al., 2012 (Japan) (22)	**Dexmedetomidine** **+** **Midazolam vs. Dexmedetomidine alone** during dental implant surgery	**Primary outcomes:** Sedation level assessed by Ramsay Sedation Score (RSS), amnesia during infiltration anaesthesia/incision/cutting/suturing, patient satisfaction. **Secondary outcomes:** Hemodynamic variables (SBP, DBP, HR), SpO_2_ changes, intraoperative arousal responses. **Results:** Group 1 (DEX 2 μg/kg/h + MDZ 0.02 mg/kg) achieved significantly deeper sedation than low-dose DEX groups. Group 4 (DEX alone) showed poorer amnesic effect during infiltration anaesthesia and incision. Group 2 showed lighter sedation with poorer evaluation in the first half of surgery. No significant difference in overall patient satisfaction.	Addition of midazolam to dexmedetomidine improved amnesia and reduced intraoperative awareness, thereby reducing anxiety and fear related to dental implant surgery. Combined sedation produced a calmer patient experience with fewer arousal episodes.

**Table 5:** Characteristics of included non-pharmacological studies.

Author and Year	Country	Study design	Sample size	Test intervention	Comparator	Outcomes evaluated
Lukkanasomboon et al., 2025 (28)	Thailand	Parallel-Group Randomized Clinical Trial	100 patients requiring implant replacement surgery	Multimedia information regarding implant surgery	Verbal explanation	Knowledge score, anxiety score, decision-making
Pellicer et al., 2024 (30)	Spain	Double-Blind Parallel RCT	78 patients requiring immediate implant placement	Baroque/Classical music	No music	SBP, DBP, HR, SpO_2_, MDAS, VAS
Ghobadi et al., 2024 (32)	Iran	Randomized Controlled Trial	73 patients undergoing two implant surgeries	Virtual reality distraction	No VR	Anxiety, pain, satisfaction, physiological responses
UcakTurer et al., 2023 (31)	Turkey	Single-Blind Parallel RCT	Patients aged 25–65 years undergoing implant surgery	Mindfulness meditation	No meditation	STAI-S, BIS, cortisol, HR, BP, SpO_2_
Pellicer et al., 2023 (29)	Spain	Randomized Controlled Clinical Trial	26 patients requiring single implant placement	Baroque/Classical music	No music	Anxiety score, pain score, BP, HR
Bertacco et al., 2022 (33)	Canada	Pilot Randomized Controlled Trial	24 dental implant surgery patients	Personalized music intervention	Audiobook control	Burden of care, anxiety, pain, dissatisfaction
Sghaireen et al., 2020 (36)	Saudi Arabia	Randomized Comparative Clinical Study	270 implant patients	Verbal information	Visual information/video	MDAS anxiety score
Camacho-Alonso et al., 2019 (34)	Spain	Parallel-Group Randomized Clinical Trial	300 patients undergoing single implant placement	Audiovisual information	Verbal information	STAI, MDAS, DFS, satisfaction
Kazancioglu et al., 2016 (35)	Turkey	Randomized Controlled Clinical Trial	60 patients undergoing implant surgery	Verbal information + surgical movie	Verbal information only/control	STAI, MDAS, VAS pain score

**Table 6:** Clinical outcomes of included non pharmacological studies.

Study	Intervention	Primary and secondary outcomes	Inference related to anxiety reduction
Lukkanasomboon et al., 2025 (28)	Multimedia information vs. verbal explanation	Knowledge score improved from **1.96** → **3.90** and anxiety reduced from **12.76** → **11.48** in multimedia group	Both multimedia and verbal education improved patient understanding and reduced preoperative anxiety
Pellicer et al., 2024 (30)	Baroque music vs. classical music vs. no music	Significant reduction in **SBP** and **MDAS anxiety scores** in music groups	Music therapy reduced physiologic stress and promoted relaxation during implant placement
Ghobadi et al., 2024 (32)	Virtual reality vs. no VR	Significant reduction in **STAI, MDAS and pain scores** with improved patient satisfaction	VR distraction effectively reduced anxiety and fear by diverting patient attention away from surgery
UcakTurer et al., 2023 (31)	Mindfulness meditation vs. no meditation	Reduced **STAI-S scores, cortisol levels, HR, SBP, DBP** and improved **SpO_2_**	Mindfulness meditation reduced both physiologic and psychologic stress during implant surgery
Pellicer et al., 2023 (29)	Baroque/classical music vs. no music	Anxiety scores significantly reduced in both music groups	Listening to music decreased anxiety and improved relaxation during surgery
Bertacco et al., 2022 (33)	Personalized music vs. audiobook	Significant reduction in burden of care, anxiety and pain memory	Personalized music created a calming environment and reduced emotional stress
Sghaireen et al., 2020 (36)	Verbal information vs. visual information	Verbal information group showed significantly lower **MDAS anxiety scores**	Face-to-face verbal explanation reduced anxiety more effectively than visual/video information
Camacho-Alonso et al., 2019 (34)	Audiovisual information vs. verbal information	**STAI, MDAS and DFS scores** significantly higher in audiovisual group	Audiovisual surgical information increased anxiety and fear before implant surgery
Kazancioglu et al., 2016 (35)	Verbal information + surgical movie vs. verbal information only	Higher **STAI and MDAS anxiety levels** observed in movie group	Multimedia surgical videos increased patient anxiety before implant procedures

### 3.3 Risk of bias

**Figure 6:**
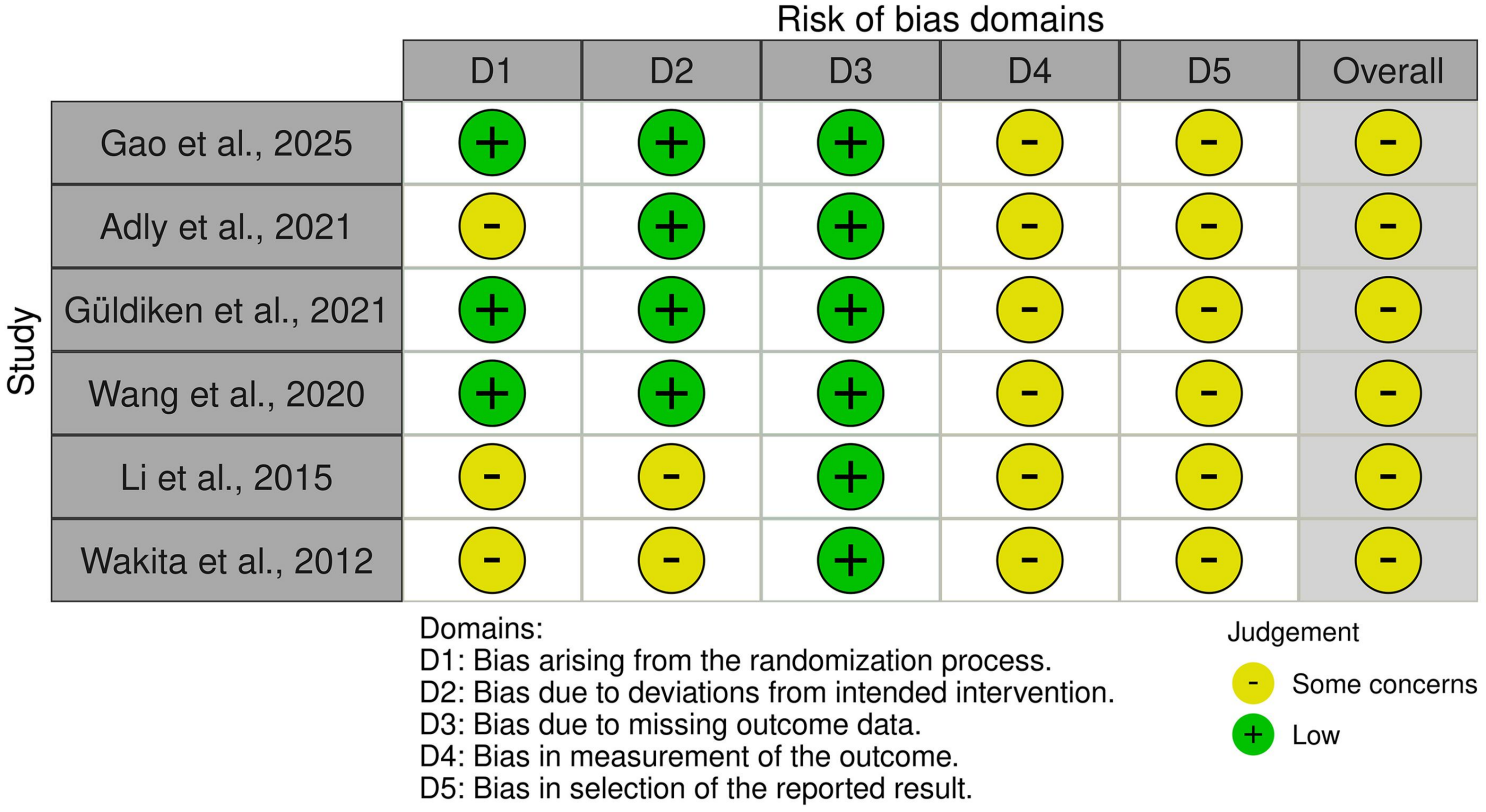
Risk of bias assessment of pharmacological intervention studies using the Cochrane RoB 2 tool.

**Figure 7:**
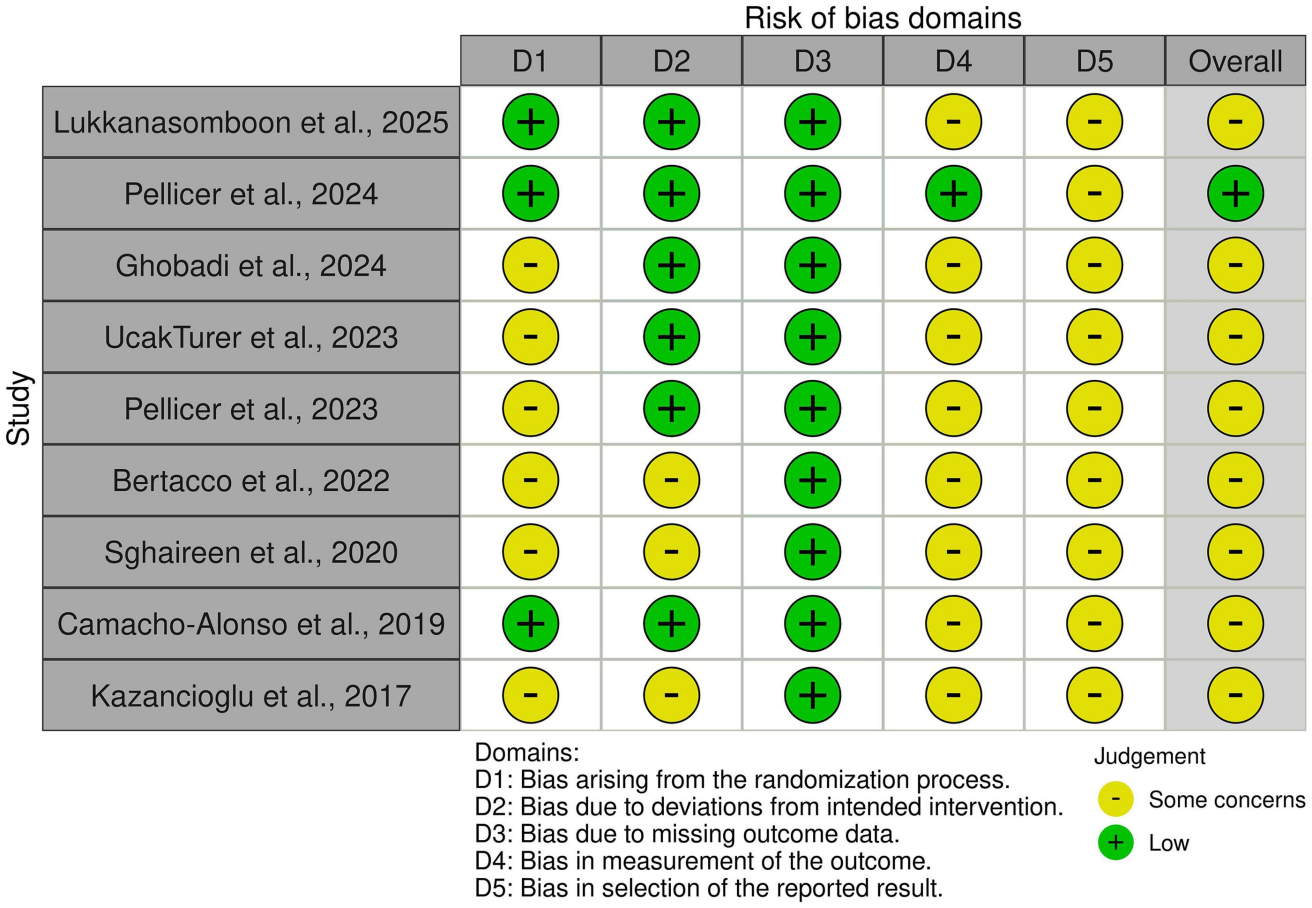
Risk of bias assessment of non-pharmacological intervention studies using the Cochrane RoB 2 tool.

Risk of bias of the 15 randomised controlled trials was assessed utilizing Cochrane Risk of Bias 2 (RoB 2) tool, in accordance with PRISMA 2020 guidelines. Risk of bias was assessed separately for each of the six randomized controlled trials on pharmacological treatment of dental anxiety (Figure 6) and the nine randomized controlled trials on non-pharmacological treatment of dental anxiety (Figure 7) included in this systematic review. Each study was evaluated across the five RoB 2 domains: (1) randomisation process, (2) deviations from intended interventions, (3) missing outcome data, (4) measurement of outcomes, and (5) selection of the reported result. Four reviewers independently analysed the risk of bias, and any discrepancies were resolved by discussion until consensus was reached.

### 3.4 Quality assessment

The objective of this systematic review was to determine the effectiveness of pharmacological and non-pharmacological interventions in reducing dental anxiety during dental implant surgery. The strength of recommendations and certainty of evidence for each intervention were assessed using the Grades of Recommendation, Assessment, Development and Evaluation (GRADE) approach. The certainty of evidence was rated as high, moderate, low, or very low based on assessments of risk of bias, inconsistency, indirectness, imprecision, and publication bias. The results of the Cochrane Risk of Bias 2 (RoB 2) assessment informed the GRADE evaluation, with evidence from studies judged to have low risk of bias being less likely to be downgraded, whereas studies with some concerns or serious methodological limitations contributed to lower certainty ratings when considered alongside the other GRADE domains. The certainty ratings for all included studies are presented in Tables 7, 8.

**Table 7:** GRADE assessment of pharmacological studies.

Study	Risk of bias	Inconsistency	Indirectness	Imprecision	Publication bias	Overall
Gao et al., 2025 (25)	Low	N/A	Not serious	Not serious	Unlikely	⊕⊕⊕⊕ High
Adly et al., 2021 (26)	Some concerns	N/A	Not serious	Serious	Unlikely	⊕⊕⊕○ Moderate
Güldiken et al., 2021 (27)	Some concerns	N/A	Not serious	Not serious	Unlikely	⊕⊕⊕○ Moderate
Wang et al., 2020 (24)	Some concerns	N/A	Not serious	Not serious	Unlikely	⊕⊕⊕○ Moderate
Li et al., 2015 (23)	Some concerns	N/A	Not serious	Not serious	Unlikely	⊕⊕⊕○ Moderate
Wakita et al., 2012 (22)	Some concerns	Serious	Not serious	Serious	Possible	⊕⊕○○ Low

**Table 8:** GRADE assessment of non-pharmacological studies.

Study	Risk of bias	Inconsistency	Indirectness	Imprecision	Publication bias	Overall
Lukkanasomboon et al., 2025 (28)	Some concerns	N/A	Not serious	Not serious	Unlikely	⊕⊕⊕○ Moderate
Pellicer et al., 2024 (30)	Some concerns	Serious	Not serious	Not serious	Possible	⊕⊕⊕○ Moderate
Ghobadi et al., 2024 (32)	Low	N/A	Not serious	Not serious	Unlikely	⊕⊕⊕⊕ High
UcakTurer et al., 2023 (31)	Some concerns	N/A	Not serious	Not serious	Unlikely	⊕⊕⊕○ Moderate
Pellicer et al., 2023 (29)	Some concerns	Serious	Not serious	Not serious	Possible	⊕⊕⊕○ Moderate
Bertacco et al., 2022 (33)	Some concerns	Serious	Not serious	Serious	Possible	⊕⊕○○ Low
Sghaireen et al., 2020 (36)	Some concerns	N/A	Not serious	Not serious	Possible	⊕⊕⊕○ Moderate
Camacho-Alonso et al., 2019 (34)	Some concerns	Consistent	Not serious	Not serious	Possible	⊕⊕⊕○ Moderate

## 4 Discussion

The evidence from 15 randomized controlled clinical trials investigating pharmacological and non-pharmacological treatments for lowering dental anxiety during dental implant surgery has been collected in this systematic review. Overall, the research that is currently available indicates that interventions like conscious sedation based on dexmedetomidine and remimazolam, music therapy, mindfulness meditation, virtual reality distraction, and structured patient education can significantly reduce anxiety, improve patient comfort, and improve the overall surgical experience during implant procedures. These results are consistent with the increased importance of individualized Preoperative care and the expanding use of multimodal anxiety-management techniques in implant dentistry.

### 4.1 Summary of observed characteristics of interventions

#### 4.1.1 Anxiety and stress reduction

Anxiety and stress reduction were the most frequently assessed outcomes in the included study. Among pharmacological interventions, Adly et al. provided the most direct evidence by evaluating salivary cortisol levels as an objective biomarker of stress which demonstrates that paracetamol significantly reduced peri operative stress and anxiety compared to ibuprofen (26). Wang et al. and Li et al. provided indirect support for the increased anxiolytic effects of dexmedetomidine over midazolam-based regimens, reporting superior sedation depth, improved patient cooperation, and greater intraoperative comfort (23, 24). Similarly, Wakita et al. highlighted the limitations of dexmedetomidine as a core and sole agent, noting episodes of intraoperative arousal and inconsistent amnesia, whereas its combination with midazolam resulted in improved sedation quality (22). Gao et al., although not primarily focused on anxiety outcomes, assessed conscious sedation in geriatric patients and concluded that both remimazolam and dexmedetomidine achieved satisfactory sedation, with remimazolam superiority offering faster onset and recovery (25).

Among non-pharmacological interventions, anxiety outcomes were evaluated more directly. Camacho-Alonso et al., Sghaireen et al., and Kazancioglu et al. consistently reported that audiovisual, visual, or video-based surgical information increased anxiety compared with conventional verbal information, suggesting that excessive procedural visualization may amplify anticipatory fear (34–36). In contrast, Lukkanasomboon et al. found that both multimedia-based and verbal education significantly reduced anxiety, with no significant difference between the two approaches, indicating that the design, content, and delivery of educational material may critically influence patient responses (28). UcakTurer et al. demonstrated that mindfulness meditation significantly reduced both State-Trait Anxiety Inventory (STAI) scores and salivary cortisol levels (31), while Ghobadi et al. reported that virtual reality effectively reduced anxiety through immersive distraction (32). Music-based interventions also showed favorable outcomes. Pellicer et al. demonstrated that baroque and classical music significantly reduced anxiety during implant placement, whereas Bertacco et al. found that personalized music reduced the overall burden of care, including anxiety-related distress (29, 30, 33). Overall, mindfulness meditation, virtual reality, and music-based interventions demonstrated more consistent anxiolytic benefits than informational interventions, which produced heterogeneous and in some cases counterproductive effects.

#### 4.1.2 Conscious sedation/sedation depth

The effectiveness of various sedative agents used during dental implant surgery has been assessed in a number of pharmacological studies. Wang et al. compared dexmedetomidine with midazolam and found that dexmedetomidine achieved significantly superior sedation, as evidenced by lower Observer's Assessment of Alertness/Sedation (OAAS) scores, along with higher surgeon satisfaction (24), patients receiving dexmedetomidine also experienced prolonged postoperative analgesia, indicating improved sedative efficacy with the dexmedetomidine-based regimen. Similarly, Wang et al. found that dexmedetomidine produced noticeably better sedation, as shown by lower Observer's Assessment of Alertness/Sedation (OAAS) scores and higher surgeon satisfaction (23, 24). Furthermore, sustained postoperative analgesia was observed in individuals taking dexmedetomidine, indicating an additional analgesic benefit beyond its sedative effects. In a similar way, Li et al. showed that the combination of dexmedetomidine and fentanyl produced deeper drowsiness and considerably lower OAAS scores when compared to the traditional midazolam–fentanyl regimen, suggesting that the dexmedetomidine-based approach had better sedative efficacy (23).

#### 4.1.3 Postoperative analgesia/pain control

Postoperative pain control was evaluated predominantly in the pharmacological studies included in this review. Wang et al. demonstrated that dexmedetomidine significantly prolonged postoperative analgesia compared with midazolam, while Li et al. reported significantly lower postoperative Visual Analog Scale (VAS) pain scores in patients receiving dexmedetomidine–fentanyl compared with the conventional midazolam–fentanyl regimen, collectively supporting the superior analgesic efficacy of dexmedetomidine-based sedation protocols (23, 24). In contrast, Adly et al. observed no statistically significant difference in postoperative pain scores between paracetamol and ibuprofen, although paracetamol was associated with greater reductions in preoperative stress, whereas ibuprofen demonstrated superior control of postoperative swelling (26). Wakita et al. primarily focused on sedation quality, amnesic effects, and patient satisfaction, without directly assessing postoperative pain as a primary outcome, while Gao et al. concentrated mainly on sedation safety, hemodynamic stability, and recovery characteristics rather than postoperative analgesic efficacy (22, 25).

Among the non-pharmacological interventions, pain-related outcomes were assessed less consistently but yielded promising findings. Ghobadi et al. demonstrated significant reductions in pain perception with virtual reality distraction, suggesting that immersive cognitive engagement may effectively modulate intraoperative pain experience during implant surgery (32). Similarly, Bertacco et al. reported that personalized music intervention significantly reduced the overall procedural burden and lowered recalled pain intensity at seven days postoperatively, whereas Pellicer et al. found that although baroque and classical music significantly reduced anxiety, no statistically significant differences in postoperative pain were observed compared with controls (30, 33). UcakTurer et al. focused primarily on anxiety, cortisol, bispectral index, and physiological stress parameters following mindfulness meditation, while informational intervention studies by Camacho-Alonso et al., Sghaireen et al., Kazancioglu et al., and Lukkanasomboon et al. mainly evaluated anxiety, knowledge, and decision-making, providing limited direct evidence regarding pain control (28, 31, 34–36). Overall, pharmacological interventions, particularly dexmedetomidine-based regimens, demonstrated the strongest evidence for postoperative analgesic benefit, while virtual reality and personalized music appear to offer promising non-pharmacological adjuncts for pain reduction.

#### 4.1.4 Patient satisfaction

Patient satisfaction was reported inconsistently across the included studies. Among pharmacological interventions, Wang et al. evaluated patient satisfaction and reported favorable outcomes with both sedative regimens however, dexmedetomidine demonstrated superior clinical performance in terms of sedation quality and analgesic efficacy (24). Similarly, Wakita et al. observed no significant difference in patient satisfaction between dexmedetomidine alone and dexmedetomidine–midazolam combination therapy, despite measurable differences in sedation depth and amnestic effects (22). Li et al. suggested that dexmedetomidine improved patient cooperation and procedural comfort, although patient satisfaction was not a primary study endpoint (23). In contrast, Adly et al. primarily focused on postoperative stress, pain, and swelling, whereas Gao et al. emphasized safety outcomes, sedation onset, and discharge readiness rather than subjective patient-reported satisfaction (25, 26).

Among non-pharmacological interventions, patient satisfaction was more prominently associated with strategies that directly enhanced the procedural experience. Ghobadi et al. reported higher patient satisfaction with virtual reality distraction, with most participants expressing willingness to use the intervention in future procedures (32). Bertacco et al. demonstrated that personalized music improved emotional well-being and reduced perceived treatment burden, indicating a more favorable patient experience (33). Camacho-Alonso et al. found that although anxiety levels were higher in the audiovisual information group, satisfaction with the information received was comparable between audiovisual and conventional verbal explanation groups (34). Lukkanasomboon et al. reported that while multimedia and verbal educational approaches improved patient knowledge, their influence on treatment decision-making was limited, suggesting that satisfaction and acceptance may be shaped more strongly by clinician guidance and economic considerations (28). Other studies, including those by Sghaireen et al. and Kazancioglu et al., primarily examined anxiety reduction following information delivery rather than patient satisfaction (35, 36). Likewise, Pellicer et al. and Ucak-Turer et al. focused predominantly on anxiety-related and physiological outcomes, with patient satisfaction not assessed as a primary endpoint (30, 31).

#### 4.1.5 Surgeon satisfaction

Surgeon satisfaction was assessed less frequently than patient-reported outcomes across the included studies. Among pharmacological interventions, Wang et al. provided the most direct evidence, reporting significantly greater surgeon satisfaction with dexmedetomidine compared with midazolam, likely attributable to improved sedation quality, enhanced patient cooperation, and superior analgesic control (24). Li et al. similarly reported better patient–surgeon cooperation with dexmedetomidine-based sedation, indirectly suggesting improved operator comfort and procedural ease (23). In contrast, Wakita et al. primarily focused on sedation depth and amnestic effects rather than surgeon satisfaction as a principal outcome (22). Adly et al. did not assess surgeon satisfaction, while Gao et al. concentrated predominantly on safety profiles, onset of sedation, and discharge readiness rather than operator-related outcomes (25, 26).

Surgeon-related outcomes were similarly limited among non-pharmacological interventions. Ghobadi et al. evaluated surgeon distress and found that virtual reality distraction reduced procedural burden from the clinician's perspective, suggesting that effective anxiety management may facilitate improved patient handling during treatment (32). Bertacco et al. assessed burden of care largely from the patient perspective rather than directly measuring surgeon satisfaction (33). Other studies, including those by Pellicer et al., Ucak-Turer et al., Camacho-Alonso et al., Sghaireen et al., Kazancioglu et al., and Lukkanasomboon et al., did not specifically evaluate surgeon satisfaction as a primary outcome (28, 31, 34–36). Overall, the available evidence suggests the strongest support for improved surgeon satisfaction with dexmedetomidine-based sedation, with limited but favorable evidence for virtual reality distraction as a supportive non-pharmacological strategy.

#### 4.1.6 Vital signs and hemodynamic changes

Vital signs and hemodynamic stability were extensively evaluated in pharmacological sedation studies. Wang et al. reported that dexmedetomidine maintained stable oxygen saturation and blood pressure, although it was associated with a lower heart rate compared with midazolam (24). Similarly, Li et al. monitored heart rate, systolic blood pressure, rate–pressure product, respiratory rate, and oxygen saturation, demonstrating that dexmedetomidine provided effective sedation and analgesia while maintaining acceptable physiological stability (23). Wakita et al. also observed reductions in heart rate and blood pressure during dexmedetomidine-based sedation, particularly at higher doses, whereas oxygen saturation remained within clinically acceptable limits (22). In a direct comparison of sedative agents in elderly patients, Gao et al. reported that remimazolam was associated with significantly less hypotension, faster onset of sedation, and shorter discharge times than dexmedetomidine, suggesting a potential hemodynamic advantage in geriatric patients undergoing implant procedures (25). In contrast, Adly et al. primarily focused on biochemical stress markers, postoperative pain, and swelling, with limited assessment of hemodynamic parameters (26).

Among non-pharmacological interventions, several studies also examined physiological outcomes related to autonomic stress responses. Ucak-Turer et al. demonstrated that mindfulness meditation significantly improved hemodynamic parameters, including reductions in heart rate, systolic and diastolic blood pressure, alongside increased oxygen saturation and reduced cortisol levels (31). Similarly, Ghobadi et al. reported that virtual reality intervention improved psychophysiological parameters, supporting its potential role in attenuating autonomic stress responses during treatment (32). Pellicer et al. observed significant reductions in systolic blood pressure among patients exposed to baroque and classical music, suggesting decreased sympathetic activation during implant surgery (30). By contrast, studies by Camacho-Alonso et al., Sghaireen et al., Kazancioglu et al., and Lukkanasomboon et al. primarily focused on anxiety, patient education, and informational outcomes, providing limited comparative evidence regarding vital signs or hemodynamic effects (28, 34–36). Bertacco et al. emphasized burden of care, emotional affect, and pain memory rather than detailed physiological monitoring (33).

Overall, pharmacological sedation studies provide more robust direct evidence regarding hemodynamic safety and physiological monitoring, whereas non-pharmacological interventions such as mindfulness meditation, virtual reality, and music therapy demonstrate promising effects in reducing physiological stress responses without the potential cardiovascular adverse effects associated with sedative agents. When pharmacological and non-pharmacological therapies were compared, pharmacological sedation approaches generated stronger and more consistent anxiety reductions, especially in highly nervous individuals or for invasive implant procedures. When compared to midazolam and traditional regimens, dexmedetomidine-based sedation consistently showed improved anxiolysis, deeper drowsiness, extended analgesia, and decreased physiologic stress indicators. On the other hand, non-pharmacological therapies were advantageous due to their non-invasiveness, affordability, and lack of sedative-related side effects. Virtual reality distraction, mindfulness meditation, and music therapy all successfully decreased mild-to-moderate anxiety and increased patient comfort without posing a pharmaceutical risk. However, by exposing patients to surgical specifics and procedural imagery, educational audiovisual treatments often raised anxiety. Thus, the evidence suggests that optimal anxiety management during dental implant surgery may require a tailored approach that combines effective pharmacological sedation for highly anxious individuals with supportive non-pharmacological strategies such as music therapy, mindfulness, and patient-centred verbal communication to enhance the overall implant treatment experience.

### 4.2 Previous research on pharmacological interventions targeted to reduce dental anxiety

Jerjes et al. (2005, United Kingdom) demonstrated that oral benzodiazepine premedication significantly reduces procedural anxiety and improves patient cooperation (37), while Coulthard et al. (2006, United Kingdom) confirmed the effectiveness of midazolam as a reliable anxiolytic with sedative and amnestic benefits (38). Similarly, Pourabbas et al. (2022, Iran) reinforced the role of conscious sedation in reducing anxiety, particularly in patients with moderate to severe dental fear (39).

Recent comparative studies have highlighted newer sedative options for improved anxiety control. Hiroyoshi Kawaai et al. (2023, Japan) found both dexmedetomidine and propofol effective, with dexmedetomidine offering smoother anxiolysis and propofol enabling faster recovery (40).

### 4.3 Previous research on non- pharmacological interventions targeted to reduce dental anxiety

Behavioral and psychological factors play a major role in dental anxiety and treatment avoidance. Corah et al. (1968, USA) and Locker et al. (1991, Canada) identified dental anxiety as a significant barrier to regular dental attendance, leading to delayed treatment and worsening oral health (41, 42). Armfield et al. (2007–2008, Australia) further described the “vicious cycle of dental fear,” were avoidance of care results in disease progression and increasingly invasive treatment, thereby intensifying anxiety (43).

Non-pharmacological interventions have shown effectiveness in managing dental anxiety, particularly in mild to moderate cases. Stephan Eitner et al. (2014, Germany) demonstrated that hypnotherapy and music-based distraction reduce preoperative anxiety, while Ganga Mohan et al. (2023, India) reported beneficial effects of yoga in reducing psychological and physiological stress during implant surgery. Supporting this, Appukuttan DP (2016, India) emphasized behavioral strategies such as effective communication, reassurance, relaxation training, distraction, and cognitive behavioral therapy as first-line approaches for anxiety (20, 44, 45).

### 4.4 Clinical implications

In order to maximize patient-centered outcomes, the results of this systematic review highlight the therapeutic significance of incorporating evidence-based anxiety management techniques into standard dental implant practice. During implant surgery, pharmacological treatments, including conscious sedation with dexmedetomidine and remimazolam, showed good analgesic and anxiolytic efficacy along with improved hemodynamic stability and higher patient-reported satisfaction. Those who have severe dental anxiety, a low pain threshold, or complicated surgical requirements may benefit most from these techniques. Furthermore, the application of suitable sedation procedures seems to reduce intraoperative stress reactions, enhance procedural effectiveness, and provide a more regulated and predictable surgical environment for both patients and clinicians.

Without the risks of further pharmacological exposure, non-pharmacological interventions like music therapy, mindfulness-based relaxation, virtual reality distraction, and structured verbal counseling have also shown clinically significant reductions in both psychological distress and physiological stress responses. These methods are useful supplements for anxiety treatment in implant dentistry because of their simplicity, affordability, and ease of inclusion into standard clinical practice. Excessive procedural visualization, especially through audiovisual records of surgical procedures, may exacerbate anticipatory anxiety and psychological discomfort in vulnerable people, according to the investigation. Therefore, in order to maximize treatment acceptability, improve patient experience, and optimize overall clinical outcomes, anxiety management strategies should be individualized and patient-centered, with interventions tailored to the patient's psychological profile, informational preferences, and procedural complexity.

### 4.5 Strengths and limitations

The present systematic review possesses several strengths, including a comprehensive evaluation of both pharmacological and non-pharmacological interventions aimed at reducing dental anxiety during dental implant surgery. The inclusion of randomized controlled trials and comparative clinical studies enhanced the methodological rigor and reliability of the synthesized evidence. Furthermore, the review incorporated a broad range of anxiety assessment methods, including validated psychometric scales and physiologic biomarkers, thereby providing a multidimensional understanding of anxiety reduction strategies. The use of the GRADE approach also strengthened the interpretability of evidence certainty and clinical recommendations. However, certain limitations must be acknowledged.

A major limitation of this review is the substantial clinical and methodological heterogeneity among the included studies. The pharmacological studies investigated a range of sedative and anxiolytic agents with different mechanisms of action, dosages, routes of administration, and sedation protocols, whereas the non-pharmacological studies evaluated diverse interventions including music therapy, virtual reality distraction, mindfulness meditation, verbal counselling, and audiovisual educational strategies. Consequently, the interventions were not directly comparable, limiting the ability to determine the superiority of any single anxiety-management approach.

Additional heterogeneity arose from differences in patient populations, implant procedures, sample sizes, anxiety severity, outcome measures, and follow-up durations. Anxiety was assessed using a variety of psychometric instruments and physiological parameters, including State—Trait Anxiety Inventory (STAI), Modified Dental Anxiety Scale (MDAS), Visual Analogue Scale (VAS), heart rate, blood pressure, oxygen saturation, salivary cortisol levels, and patient satisfaction scores. Such variability complicates cross-study comparisons and may partly explain inconsistencies observed in treatment effectiveness.

Importantly, the observed heterogeneity reflects the multifactorial nature of dental anxiety and the absence of a standardized approach for anxiety assessment and management in implant dentistry. This diversity enhances the external relevance of the findings to real-world clinical practice. A quantitative meta-analysis was not performed because of substantial clinical and methodological heterogeneity among the included studies. Therefore, the results should be interpreted as evidence supporting the effectiveness of multiple anxiety-management strategies rather than definitive proof of the superiority of one intervention over another. Future research should focus on standardized outcome measures and direct head-to-head comparisons of the most promising pharmacological and non-pharmacological approaches to strengthen the evidence base for clinical decision-making.

### 4.6 Future research directions

To strengthen the quality, comparability, and clinical applicability of the evidence base surrounding anxiety-management interventions in dental implant surgery, future research should prioritize well-designed multicentre randomized controlled trials with larger sample sizes, robust methodological frameworks, and standardized outcome measures. Incorporating validated psychometric assessment instruments like the State-Trait Anxiety Inventory (STAI) and the Modified Dental Anxiety Scale (MDAS) with objective physiological biomarkers will improve methodological rigor and enable more uniformity across investigations. To assess the long-term impacts of pharmaceutical and non-pharmacological therapies on patient satisfaction, oral health-related quality of life, treatment acceptability, and readiness to have additional dental procedures, longitudinal studies are also necessary. Furthermore, emerging patient-centered technologies, including immersive virtual reality platforms, digital mindfulness applications, biofeedback-based interventions, and multimodal integrative approaches, should be systematically explored as potentially effective and minimally invasive strategies for optimizing anxiety reduction in implant dentistry.

## 5 Conclusion

Both pharmacological and non-pharmacological interventions were associated with reductions in dental anxiety during dental implant surgery. Pharmacological approaches, including conscious sedation protocols involving dexmedetomidine, showed favorable anxiolytic outcomes in several studies, particularly among patients with moderate to severe dental anxiety. Similarly, non-pharmacological interventions such as music therapy, virtual reality distraction, mindfulness-based techniques, and structured verbal communication were associated with reduced anxiety levels and improved patient experiences, especially in individuals with mild to moderate anxiety. However, the included studies exhibited substantial clinical and methodological heterogeneity with respect to intervention protocols, patient characteristics, anxiety assessment methods, outcome measures, and follow-up periods. In addition, the absence of a quantitative meta-analysis limited the ability to directly compare interventions or determine their relative effectiveness. Therefore, the current evidence supports the potential usefulness of both pharmacological and non-pharmacological strategies for managing dental anxiety during implant therapy, but the certainty of the evidence remains limited. Conclusions regarding the superiority of any specific intervention, including dexmedetomidine-based approaches, should be interpreted with caution. Further high-quality randomized controlled trials using standardized outcome measures and direct comparative designs are needed to strengthen the evidence base and inform clinical recommendations for anxiety management during dental implant treatment.

## Data Availability

The original contributions presented in the study are included in the article/Supplementary Material, further inquiries can be directed to the corresponding author.
